# Effectiveness of Amlexanox and Adcortyl for the treatment of recurrent aphthous ulcers

**DOI:** 10.4317/jced.52540

**Published:** 2016-10-01

**Authors:** Farid Abbasi, Maryam Raoof, Roya Khatami, Niloofar Shadman, Farnaz Borjian-Boroojeni, Farahnaz Nazari

**Affiliations:** 1 Associate Professor. Department of Oral Medicine, School of Dentistry, Shahed University of Medical Sciences, Tehran, Iran; 2 Assistant Professor. Laboratory of Molecular Neuroscience, Neuroscience Research Center, Institute of Neuropharmacology, Kerman University of Medical Sciences, Kerman, Iran; 3 Postgraduate Student of Oral Medicine. School of Dentistry, Shahed University, Tehran, Iran; 4 Assistant Professor. Department of Operative Dentistry, School of Dentistry, Kerman University of Medical Sciences, Kerman, Iran; 5 Department of Oral Medicine, School of Dentistry, Shahed University, Tehran, Iran; 6 Postgraduate Student of Endodontics. School of Dentistry, Kerman University of Medical Sciences, Kerman, Iran

## Abstract

**Background:**

Recurrent aphthous stomatitis (RAS) is a common condition that affects approximately 20% of the general population. The ulcers can interfere with speech and eating and cause significant pain and discomfort. This study aimed to evaluate the efficacy of Amlexanox and Adcortyl in the treatment of aphthous ulcers.

**Material and Methods:**

In this randomized double blind clinical trial with sequential patient entry, a total of 40 patients who presented with aphthous ulcers were included. Patients were received Amlexanox or Adcortyl four times daily for 7 days. Patients were evaluated for pain, lesion size, and tingling at one day, three days, five days and seven days follow-ups. The treatment effects were then evaluated using the Wilcoxon–Mann–Whitney (WMW) test. Values of *p*<0.05 were considered significant.

**Results:**

No significant differences in pain score, tingling and lesion size were observed on similar days between Amlexanox and Adcortyl groups. In both groups, reduction in the assessed variables was significant between days 1-3, 3-5, and 5-7 (*p* < 0.001).

**Conclusions:**

This study indicated that Amlexanox as well as Adcortyl was effective in relieving pain and reducing the lesion size during the treatment of aphthous ulcers.

** Key words:**Recurrent aphthous stomatitis, Amlexanox, Adcortyl, pain relief.

## Introduction

Recurrent aphthous stomatitis (RAS) is a common condition that characterized by recurrent attacks of single or multiple small pin-head ulcers covering by fibrin on the mucous of mouth and rarely genital region that affects approximately 5-25% of the general population (1).

This condition is associated with recurrent bouts of single or multiple rounded, shallow and painful lesions surrounded by inflammation in oral mucosa that may occur at intervals of few days to a few months. These lesions most commonly occur on the non-keratinized mobile oral mucosal surfaces and heal within 10-14 days (2,3).

Studies have introduced different factors that predispose to RAS, such as systemic diseases, genetic predisposition, immune disorders, drugs, stress, trauma, foods, hormonal changes, nutrients deficiency, and so on. However, the etiology of RAS still remains unknown (4-6).

Pain is the obvious characteristic of the aphthous ulcerations causing difficulty while chewing, swallowing, and speaking (2). So far, a number of different treatment options have been introduced such as steroids, analgesics, topical anesthetics agents, antiseptics and anti-inflammatory agents, sucralfate suspension, tetracycline suspension, silver nitrate cauterization, laser ablation and traditional and home complementary remedies. Because of the broad spectrum of side effects following administration of some agents including steroids, the topical drug forms such as pasts are preferred. However, there is no definitive curative treatment for RAS (7-9).

In this regard, some studies have been conducted on the use of Amlexanox as an anti-inflammatory antiallergic immunomodulator drug to treat recurrent aphthous ulcers (10-12). According to our knowledge, no study has been carried out in Iran on the effects of either Amlexanox or Adcortyl in the treatment of aphthous ulcers.

On the other hand, the identification of the relation between genotype and drug response, including both the therapeutic effect and side effect profile, is expected to deeply affect medical practice (13). So, the purpose of the present study is to evaluate the effectiveness of Amlexanox and Adcortyl for the treatment of recurrent aphthous ulcers in Iran.

## Material and Methods

This double-blinded randomized clinical trial was approved by the research ethics committee at Shahed University of Medical Sciences, Tehran, Iran. The inclusion criteria for patients bearing recurrent aphthous ulcers were as follows: age between 18 and 40 years old and above, having minor aphthous ulcer not more than 2 days, not taking any analgesic, antiseptic or systemic or topical corticosteroid therapy prior to the study. Exclusion criteria were patients with systemic diseases (ulcerative colitis, Crohn’s disease, and Behcet disease), smoking, pregnancy or lactating, undergoing therapy for aphthous ulcers, wearing a denture, estimation of poor cooperation during the study. Each patient was informed about the procedure and his/her informed consent was obtained.

Forty patients attending Shahed Dental School and Firoozgar hospital were randomized into two groups equally. In group 1, Adcortyl 0.1% was administered, while the group 2 received Amlexanox. The patients used 5% Amlexanox four times daily for 7 days. The application of drugs was instructed to the subjects and they were asked to use it after every meal and before sleep. The patients were also asked to refrain from using any other medications. The treatment duration was 7 days and patients were asked to evaluate the severity of pain and tingling by using a 10-point visual analog scale (VAS: 0-10; 0=no pain or tingling and 10= worst possible pain or tingling). The scores were assessed at one day, three days, five days and seven days follow-ups. The area (mm2) of ulceration was measured by a caliper. The clinician who performed assessments and patients were unaware of the treatment.

-Statistical analysis

The collected data were analyzed by SPSS version 17.0. Results were compared using t-test, Wilcoxon and ANOVA with turkey’s as post hoc tests. The statistically significant level was accepted as a *p* value < 0.05.

## Results

The study group comprised of 18 men and 22 women with an average age of 36.2±3.36 years. The groups were similar in terms of age, gender, referral day, lesion size, pain and tingling at admission. No side effect, which could restrict the progression of treatment, was reported by the cases who applied the study drugs and all participants finished the period of the study.

**Figure 1:**
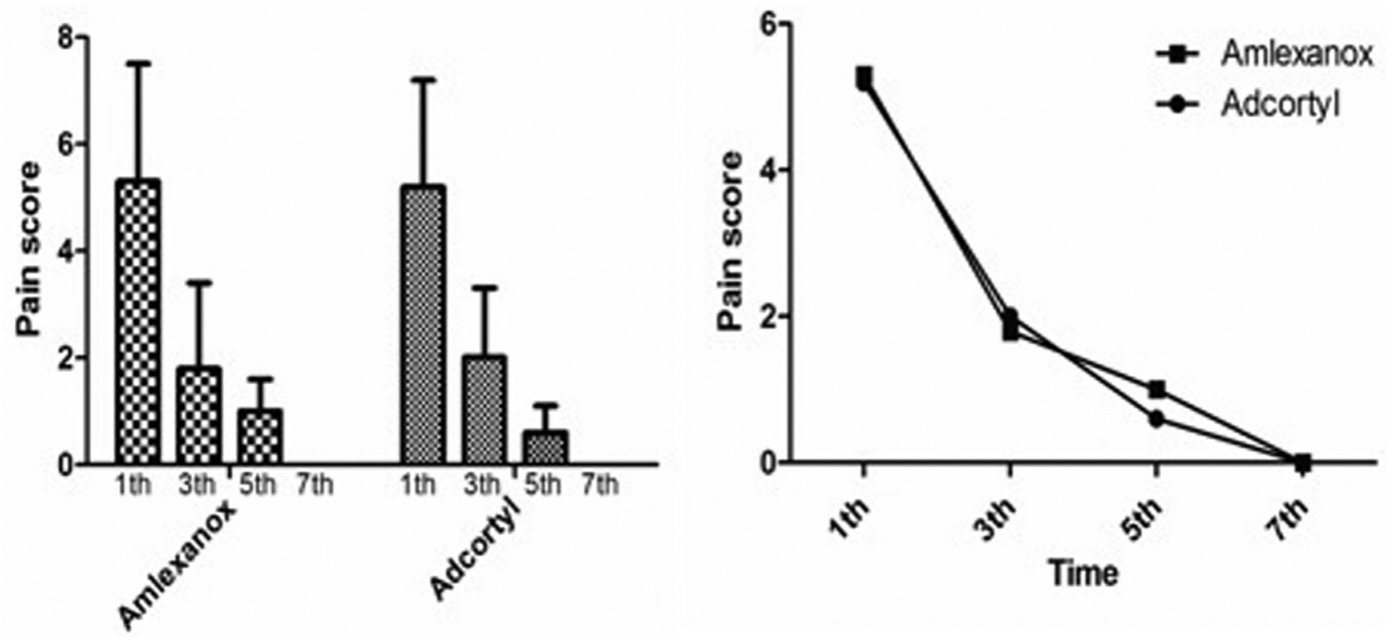
The mean value of pain scores on days 1, 3, 5 and 7 of the treatment in Amlexanox and Adcortyl groups. There were statistically significant differences between the studied time intervals in both of the groups (*p* < 0.001) (left). Trend of pain intensity based on VAS on days 1, 3, 5 and 7 of the treatment in both of the groups. There was no significant difference between the Amlexanox and Adcortyl group for pain reduction (right).

The results revealed that in both of the groups, the pain score was significantly reduced at subsequent follow-ups at 1st, 3d, 5th and 7th days (*p*<0.001). At 7 days, none of the patients in both groups experienced pain. There were no significant differences between Adcortyl and Amlexanox in terms of their efficacy on pain relieve observed in the present study (Fig. 1).

**Figure 2:**
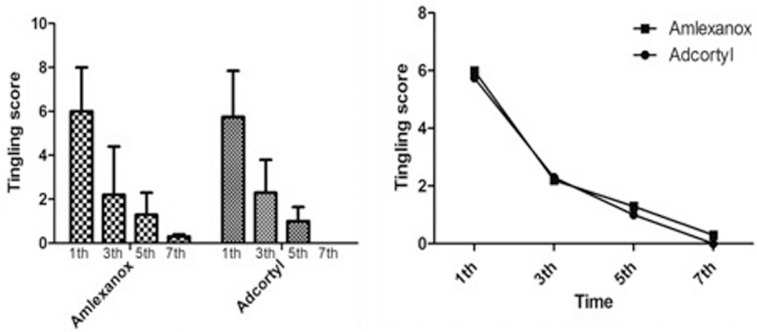
The mean value of tingling scores on days 1, 3, 5 and 7 of the treatment in Amlexanox and Adcortyl groups. There were statistically significant differences between the studied time intervals in both of the groups (*p* < 0.001) (left). Trend of tingling intensity based on VAS on days 1, 3, 5 and 7 of the treatment in both of the groups. There was no significant difference between the Amlexanox and Adcortyl groups for tingling reduction (right).

Moreover, both of the drugs led to remarkable reduction in tingling score at subsequent follow-ups at 1st, 3d, 5th and 7th days (*P*<0.001). Interestingly, despite the participants in Amlexanox group, the patients that used Adcortyl didn’t report any tingling at the 7th day (Fig. 2 left). However, there were no significant differences between Adcortyl and Amlexanox in terms of their efficacy on tingling relieve observed in the present study (Fig. 2).

**Figure 3:**
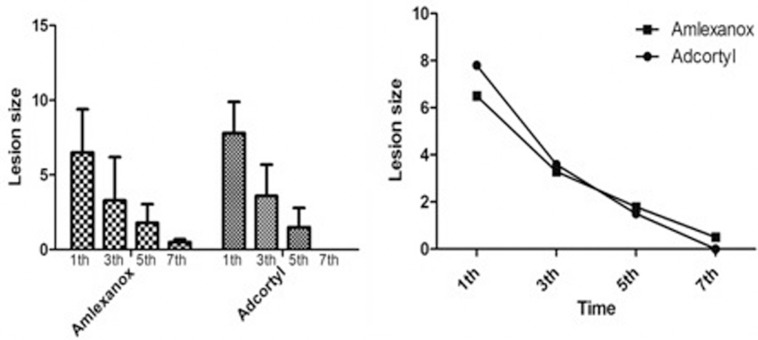
The mean ulcer size (mm2) on days 1, 3, 5 and 7 of the treatment in Amlexanox and Adcortyl groups. There were statistically significant differences between the studied time intervals in both of the groups (*p* < 0.001) (left). Trend of ulcer size on days 1, 3, 5 and 7 of the treatment in both of the groups. There was no significant difference between the Amlexanox and Adcortyl groups for reduction of ulcer size (right).

Based on the results of Wilcoxon test, lesion size in each of the groups was significantly decreased at subsequent follow-ups at 1st, 3d, 5th and 7th days (*p*<0.001). However, there were no significant differences between the two groups in terms of lesion size at 1st, 3d, 5th and 7th day follow-ups (Fig. 3 right). Despite the Amlexanox group, the score for lesion size in Adcortyl group was zero at the 7th day (Fig. 3).

## Discussion

RAS is characterized by remarkably painful ulcers causing difficulty in eating, swallowing, and speaking. The main goal of treatment modality is to decrease pain, healing time, number and size of the ulcer (14). The present study showed that both Amlexanox and Adcortyl decreased pain intensity, tingling and size of ulcers within 7 days. Moreover, there were no significant differences between the groups.

Consistent with the present results, Liu J *et al.* (10) have reported that Amlexanox significantly reduces the pain severity of aphthous stomatitis. Khandwala A *et al.* (15) stated that in comparison to vehicle-treated subjects, the subjects treated with 5% Amlexanox expressed greater reduction in ulcer size and pain on days 3 to 5. A clinical study had compared the recurrence rate between the Amlexanox and control group and proved that 5% of Amlexanox oral paste is clinically beneficial in reducing the pain, erythema, exudation and size of the ulcer over a period of 6 days (16). Murray B *et al.* (11) concluded that treatment with 5% Amlexanox paste at the onset of prodromal recurrent minor aphthous ulceration symptoms can prevent progression to ulcer development and significantly reduced symptoms if ulcers do develop. In a study, Natah SS *et al.* (17) concluded that the best treatment for aphthous minor might be 5% Amlexanox with multiple actions including preventing recurrence, decreasing healing time and pain reduction. It’s especially true when it is used from the prodromal stage till healing completes for four times a day.

Amlexanox is currently the only clinically proven product approved by the US FDA for the treatment of aphthous ulcers (18). Its mechanism of action is not well determined, but Amlexanox have anti-allergic and anti-inflammatory properties. These activities may be important in accelerating the healing process (19).

Due to the side effects and adverse events of systemic drugs, local drug delivery systems have been widely developed regarding the treatment of oral lesions (20). This may be an advantage of the paste form of Amlexanox. However, it has been shown that most of the systemic absorption of Amlexanox probably results from the gastrointestinal track after swallowing the 5% Amlexanox paste rather than directly through the ulcer. On the other hand, in the safety and efficacy studies of 5% Amlexanox paste for the treatment of aphthous ulcers, the incidence of adverse events potentially related to 5% Amlexanox paste was very low and similar to the vehicle (21). On the other hand, the long -term use of Adcortyl has been reported to be associated with the development of local candida infection (22).

We had limitations in this study in regard to relatively small sample size and duration of the study period, which require future randomized controlled trial studies in larger populations. Since aphthous has recurrent characteristics, so the importance of long-term treatment program may be beneficial.

In conclusion, it seems that both Amlexanox and Adcortyl are effective treatment options for RAS. However, there were no significant difference between Amlexanox and Adcortyl for treatment of RAS regarding pain, tingling and ulcer size reduction.
